# Fatal Pediatric Meningococcal Invasive Disease Caused by Neisseria meningitidis Serogroup C and Co-Infected With SARS-CoV-2: Report of a Case in Tijuana, Mexico

**DOI:** 10.7759/cureus.22100

**Published:** 2022-02-10

**Authors:** Enrique Chacón-Cruz, Erika Z Lopatynsky, Jesus R Machado-Contreras, Ricardo Gatica-Herrera, Oscar E Zazueta

**Affiliations:** 1 Infectología, Hospital General de Tijuana, Tijuana, MEX; 2 Health-State Scientific Committee, Secretaria de Salud de Baja California, Mexicali, MEX; 3 Family Medicine and Public Health, University of California San Diego, San Diego, USA; 4 Laboratorio de Patogenesis Molecular, Facultad de Medicina, Universidad Autonoma de Baja California, Mexicali, MEX; 5 Pediatria, Hospital General de Tijuana, Tijuana, MEX; 6 Epidemiologia, Secretaria de Salud de Baja California, Mexicali, MEX

**Keywords:** vaccine-preventable diseases, co-infection, sars-cov-2, covid-19, invasive meningococcal disease, meningococcal disease

## Abstract

Invasive meningococcal disease (IMD) is a severe infection caused by *Neisseria meningitidis*, with mortality rates ranging from 10% to 40%. IMD has been confirmed to be an endemic disease in Tijuana, Mexico, right across the border from San Diego, California. To date, coronavirus disease 2019 (COVID-19) is the most severe pandemic, causing more than 5.5 million deaths globally. Prior or co-infections of influenza with IMD has been reported previously; however, the participation of other respiratory viruses facilitating the invasiveness of *N. meningitidis* is either not shown or remains unclear. Here, we report the case of an unvaccinated (for IMD and COVID-19) seven-year-old child who had confirmed fatal IMD caused by *N. meningitidis*, serogroup C, and was co-infected by severe acute respiratory syndrome coronavirus 2.

## Introduction

Invasive meningococcal disease (IMD) is a potentially lethal infectious illness caused by *Neisseria meningitidis* into the bloodstream causing septicemia, and potentially leading to severe meningitis, acute purpura, and, less frequently, arthritis, conjunctivitis, pleural effusion, and pericarditis, among others. It is potentially fatal within 24 hours of the onset of illness and has an average case fatality ratio ranging from 10% to as high as 40% [1-4]. In addition, among survivors, up to 20% may experience permanent or long-term sequelae [2]. Clinical symptoms often start as non-specific but can progress into more specific signs of meningitis or sepsis during the infection [1].

Globally, the incidence rates of IMD vary from less than one case per 100,000 individuals (in 2016, in Europe and the United States) [1-4] to 10-1,000 cases per 100,000 individuals (in the epidemic regions of sub-Saharan Africa) [1,4]. It predominantly affects infants and young children, followed by a smaller peak in adolescents and young adults, with the latter group considered the predominant nasopharyngeal carrier age group [1-4].

In Mexico, IMD is considered a rare entity based on national reports, with an estimated national annual incidence of 0.056/100 cases based on passive surveillance [5]. However, recently published hospital-based active-prospective surveillance studies have shown that IMD is endemic in certain areas of northern Mexico, particularly in Tijuana (across the border from San Diego, California, USA). Annual incidence rates of IMD have been to be as high as 7.61 per 100,000 in children younger than two years [6-8].

In Mexico, meningococcal vaccination is not part of the national immunization program (NIP), and it is only available through the private sector. Therefore, IMD remains a potential threat for all vulnerable populations.

Coronavirus disease 2019 (COVID-19) has been described as the most important global pandemic in the last century, causing almost 350 million cases and 5.6 million deaths globally as of January 22, 2022 [9]. In Mexico, more than 4 million cases have been reported, with more than 300,000 deaths as of January 11, 2022 [10].

Influenza infection has been previously associated with IMD, even triggering the latter [11,12]. However, other respiratory viruses, such as respiratory syncytial virus (RSV) and adenovirus, have either not or only suggestively been associated with IMD [13-15]. There is only one published case from Scotland of a young adult IMD patient co-infected with severe acute respiratory syndrome coronavirus 2 (SARS-CoV-2), resulting in a good outcome [16].

This report presents the first case of a child with IMD caused by *N. meningitidis* serogroup C who was co-infected with SARS-CoV-2, which unfortunately resulted in a fatality.

## Case presentation

We present the case of a seven-year-old male with no prior clinically relevant history. Additionally, there was no personal or family history of immunodeficiencies. His older brother and father were healthy; however, his mother had died two days before our patient’s onset of symptoms due to an apparent acute fulminant clinical purpura. No studies or autopsy were performed to establish her diagnosis, and she only received unspecified antibiotics and antipyretics. We were unable to obtain more information.

Three days before admission, our patient developed fever and headache. On the second day, he developed vomiting and was taken to a private family physician who prescribed oral amoxicillin and paracetamol. Later, he developed skin lesions (described below), followed by neck stiffness. He presented to our hospital on day four of illness. On presentation, respiratory symptoms suggestive of COVID-19, such as coughing, rhinorrhea, sneezing, and anosmia, were denied during the initial interrogation.

On admission, the patient was unconscious, with a fever of 38.9°C, blood pressure of 70/40 mmHg, tachycardia, polypnea, and lung auscultation was negative for crackling rales. Multiple purpuric and petechial lesions were noted in all four limbs, back, abdomen, and thorax. The neurologic evaluation showed neck stiffness and a Glasgow Coma Scale score of seven. Clear signs of capillary leakage led to immediate intubation, mechanical ventilation, and the use of vasopressors in the intensive care setting.

We immediately started antimicrobial therapy with intravenous (IV) ceftriaxone (100 mg/kg/day) and IV doxycycline (4.4 mg/kg/day) to cover both meningococcal and rickettsial diseases.

His laboratory findings at the time of admission and after eight hours are shown in Table 1, all of which showed clear evidence of septic shock, disseminated intravascular coagulation, metabolic acidosis, and multisystemic failure. Additionally, a chest X-ray was performed on admission and was normal. The patient died nine hours following admission. Soon after he passed, we performed a spinal tap for cerebrospinal fluid (CSF) analysis, and results showed clear signs of acute neutrophilic meningitis. A Gram stain showed Gram-negative diplococci (Table 1). However, both blood and CSF cultures were negative, most likely because the patient received oral and IV antibiotics.

**Table 1:** Laboratory findings.

Laboratory results	December 30, 2021	December 31, 2021
Blood		
Hemoglobin (g/dL)	12	8
Leukocytes (total per field)	22,250	30,860
Neutrophils (%)	93%	92%
Lymphocytes (%)	2.5%	3.5%
Platelets (total)	36,670	27,560
Prothrombin time (seconds)	16.3	18.5
Partial thromboplastin time	45.4	67.8
Fibrinogen (mg/dL)	249	176
Procalcitonin (ng/mL)	23.82	43.4
D-dimer (ng/mL)	13493	14,267
Glucose (mg/dL)	82	91
Creatinine (mg/dL)	1.19	1.2
Blood urea nitrogen (mg/dL)	42	57
Alanine aminotransferase (U/L)	129	96
Sodium (mmol/L)	128	128
Potassium (mmol/L)	4.2	5.2
Chloride (mmol/L)	96	102
Albumin (g/dL)	2.7	2.2
pH	7.2	7.12
Lactate (mmol/L)	7.5	8.5
Bicarbonate (mmol/L)	12	10
Cerebrospinal fluid		
Glucose (mg/dL)		8
Proteins (g/dL)		360
Leukocytes (total per field)		1,370
Neutrophils (%)		99%
Gram stain		Gram-negative diplococcus
Culture		Negative
Polymerase chain reaction		Positive for N. meningitidis Serogroup C

A reverse transcription-polymerase chain reaction (RT-PCR) (QIAGEN®, Shanghai, China) test from CSF was performed and was positive for serogroup C, *N. meningitidis*. Moreover, as part of the hospital’s policies and regulations of performing tests for COVID-19 for all patients admitted with some respiratory symptoms, we performed a nasopharyngeal swab for RT-PCR testing of SARS-CoV-2 (2019-nCoV Centers for Disease Control and Prevention (CDC) kit, Integrated DNA Technologies®, Coralville, IO, USA), influenza (A and B) and RSV (Allplex Respiratory Panel 1A for detection of Flu A (H1, H3, H1 pdm09), Flu B, RSV A, RSV B, Seegene®, Seoul, Korea). Accordingly, the RT-PCR test for SARS-CoV-2 was positive but negative for Influenza and RSV. Furthermore, indirect immunofluorescence assay in serum was negative for Rickettsia species.

Patients’ immunoglobulins and complement in sera were normal. Enzyme-linked immunosorbent assay (ELISA) for human immunodeficiency virus was negative. Regarding contacts, all in-house and close contacts based on the current CDC guidelines received ciprofloxacin (>18 years old) or rifampin (<18 years of age); no vaccination was implemented, the latter as a decision from local health authorities. No autopsy was performed due to local COVID-preventive measures in our hospital; however, IMD and SARS-CoV-2 infection were confirmed.

## Discussion

Several predisposing conditions have been associated with IMD, such as young age; immunocompromising diseases (particularly humoral or complement deficiencies); climate factors including Harmattan and Santa Ana winds in Sub-Saharan Africa, and Tijuana, Mexico, respectively; and prior or concurrent influenza infection, among others [1,2,11,12,17].

Our patient was previously healthy with no apparent immunodeficiency. Both serum immunoglobulins and complement levels were normal. In addition to a negative ELISA for HIV, all of which indicated that he was not immunocompromised.

Particularly regarding influenza, its effect on the respiratory mucosa has been examined. It causes lysis of epithelial cells and other local immunologic damages, enhancing the invasiveness of pathogenic bacteria such as *N. meningitidis*. RSV has been shown not to affect or influence IMD, and other respiratory viruses have not consistently proven to enhance the invasiveness of *N. meningitidis* [11-15].

On the other hand, the role of community-acquired bacterial co-infection with SARS-CoV-2 has been characterized, however, mainly as a complication of COVID-19 pneumonia and not very important as secondary bacteremia [18,19]. Our patient never had any respiratory symptoms, and maybe neither her mother presumably died of IMD and was also infected with SARS-CoV-2.

There is only one report of a 22-year-old woman in Scotland who developed a co-infection of IMD and COVID-19. The patient was admitted and treated promptly, resulting in a favorable outcome. However, the latter cannot be inferred because of her vaccination against IMD as the report does not mention which meningococcal serogroup caused it. However, the authors do note that the patient was vaccinated for *N. meningitidis* A, C, Y, W during childhood, but not against serogroup B [16].

Our patient was vaccinated against IMD and COVID-19 because universal meningococcal vaccination is not part of the Mexican National Immunization Program. Moreover, in Mexico, COVID-19 vaccination for children younger than 12 years has not been implemented yet [20].

We presented the first fatal case of a child who developed IMD while co-infected with SARS-CoV-2. Whether the latter influenced *N. meningitidis* invasiveness remains unknown, and hence, we cannot infer any association. Previous publications are conclusive that universal meningococcal vaccination should be part of Tijuana’s immunization program. In addition, children should be considered for vaccination against COVID-19 in Mexico to prevent cases and hospitalizations and help avoid the spread of the pandemic.

## Conclusions

IMD is a potentially lethal disease, particularly in children, mainly in the unvaccinated population. COVID-19 is the most devastating pandemic in the last 100 years. Even though co-infection with pathogenic bacteremia has not been substantially linked, the risk of invasive bacterial diseases is present. The fatal outcome of our patient could have been avoided if meningococcal and COVID-19 vaccinations were administered. However, it remains unclear whether SARS-CoV-2 co-infection in our patient with fatal IMD increased meningococcal virulence. Nevertheless, we should expect this association to continue in the future. Active surveillance for both COVID-19 and IMD infections is essential to identify future co-infections in our region.
